# Metabolic Profiling of Distinct *TP53*-Mutant Esophageal Adenocarcinoma Models Reveals Different Bioenergetic Dependencies

**DOI:** 10.3390/ijms26146869

**Published:** 2025-07-17

**Authors:** Erica Cataldi-Stagetti, Nicola Rizzardi, Arianna Orsini, Bianca De Nicolo, Chiara Diquigiovanni, Luca Pincigher, Noah Moruzzi, Romana Fato, Christian Bergamini, Elena Bonora

**Affiliations:** 1 Department of Medical and Surgical Sciences (DIMEC), University of Bologna, 40126 Bologna, Italy; erica.cataldi2@unibo.it (E.C.-S.); arianna.orsini8@unibo.it (A.O.); bianca.denicolo2@unibo.it (B.D.N.); chiara.diquigiovanni@unibo.it (C.D.); 2 Department of Pharmacy and Biotechnology, University of Bologna, 40126 Bologna, Italy; nicola.rizzardi2@unibo.it (N.R.); luca.pincigher2@unibo.it (L.P.); romana.fato@unibo.it (R.F.); 3 Piattaforma Medicina e Biologia Molecolare, IRCCS Azienda Ospedaliero, Universitaria di Bologna, 40126 Bologna, Italy; 4 Department of Molecular Medicine and Surgery, K1 MMK Signaltransduktion, 171 77 Stockholm, Sweden; noah.moruzzi@ki.se

**Keywords:** esophageal adenocarcinoma, metabolism, *TP53*, mitochondria

## Abstract

Esophageal adenocarcinoma (EAC) is a highly aggressive malignancy with rising incidence and poor prognosis. *TP53*, previously identified as the most frequently mutated gene in EAC in our studies, plays a central role in tumor suppression and regulation. However, the metabolic consequences of *TP53* mutations in EAC remain largely uncharacterized. We metabolically profiled three *TP53*-mutant EAC cell models (OE33, OE19, and FLO1) representing progressive stages of tumor differentiation and harboring distinct *TP53* alterations. Our analyses revealed different metabolic phenotypes associated with *TP53* status. OE33 cells predominantly use glycolytic metabolism but display limited adaptability to environmental changes, possibly due to a higher differentiation state. FLO1 cells exhibit a strong glycolytic dependence, elevated lactate production, and robust proliferation under acidic conditions, consistent with an aggressive and metastatic phenotype. OE19 cells preferentially utilize oxidative phosphorylation, demonstrated by resilience to glucose and glutamine deprivation, and ROS accumulation. These findings highlight the metabolic plasticity of EAC and suggest that *TP53* mutation type might influence bioenergetic dependencies. Targeting these metabolic vulnerabilities may offer novel therapeutic avenues for personalized treatment in EAC.

## 1. Introduction

Esophageal adenocarcinoma is one of the most lethal malignancies worldwide, characterized by high aggressiveness and mortality rates [1]. The incidence of EAC is rising in Western countries, with projections suggesting that, by 2030, 1 in 100 men in the UK and the Netherlands may be diagnosed with EAC [2].

Through omics analysis, several studies have shown that EAC is characterized by high chromosomal instability and mutation rates compared with other types of cancer [3,4]. Genome instability in EAC includes genomic catastrophes [5,6], with telomere shortening [7], complex rearrangements, breakage–fusion–bridge (BFB), localized hypermutations defined as kataegis (characterized by groups of C>T and C>G mutations on the same strand), and chromotripsis [6]. Recent studies have unveiled how chromothripsis can promote tumorigenesis by driving the activation of oncogenes and the silencing of tumor suppressors [8,9]. A high rate of copy number alterations (CNAs) is possibly correlated with worse outcomes [10]. Through a combination of methods for identifying recurrent alterations, i.e., high-impact functional mutations and amplification or deletion of genomic regions, 76 driver genes were identified, including *TP53* (72%), *CDKN2A* (28%), *KRAS* (19%), *MYC* (19%), *ERBB2* (18%), *GATA4* (15%), *SMAD4* (15%), *CCND1* (14%), *GATA6* (14%), *CDK6* (14%), *ARID1A* (13%), and *EGFR* (12%) [11]. Despite these findings, the use of these complex molecular data to stratify patients effectively and guide clinical decision-making remains a significant challenge [12].

*TP53* is the most frequently mutated gene in EAC, and its alteration is considered an early genetic event in EAC cancer progression [13,14]. p53 is essential for cell cycle control and the induction of apoptosis in response to DNA damage [15]. We recently compared the effects of different *TP53* mutations (missense changes vs. loss-of-function) in a cohort of treatment-naïve EAC cases in terms of correlation with histological and clinical data [16]. The presence of pathogenic missense mutations in *TP53* was correlated with poor cancer-specific survival in cases classified as high risk according to the EACSGE classification, based on morphological features and tumor staging [17]. Therefore, *TP53* mutation status may serve as a biomarker for prognosis and for guiding the selection of the most effective targeted therapy strategies [16].

In this study, we evaluated the metabolic consequences of different *TP53* mutations in three EAC cell models, OE33, OE19, and FLO1, which represent distinct stages of tumor differentiation and harbor missense or loss-of-function mutations in *TP53*. The OE33 cell line is characterized by a high degree of differentiation and represents tumor stage IIA, according to the UICC classification. The OE19 cell line represents a stage III model. FLO1 cells correspond to an aggressive and metastatic phase of tumor stage III. At the genetic level, OE33 and FLO1 cells harbor missense variants in the DNA-binding domain of p53 (c.404 G>A, p.Cys135Tyr and c.830 G>T, p.Cys277Phe, respectively), whereas OE19 cells carry a frameshift variant in the tetramerization domain (c.929dupA, p.Asn310Lysfs*27) that severely disrupts p53 functionality. In addition, this cell line carries an amplification of the *ERBB2* gene, an event that is frequently present in association with *TP53* mutations in EAC patients [18].

p53 integrates cellular signals from various stress conditions, such as DNA damage, hypoxia, and metabolic and replicative stress, triggering protective responses including apoptosis, senescence, cell cycle arrest, and DNA repair [19,20,21,22]. Moreover, p53 plays a fundamental role in maintaining cellular homeostasis even in the absence of overt stress, dynamically regulating cell physiology [23]. However, the impact of *TP53* mutations on the metabolic landscape of EAC remains largely unexplored. To address this knowledge gap, we metabolically characterized the three p53-mutant EAC cell lines to uncover potential metabolic vulnerabilities that could provide additional therapeutic targets for EAC treatment.

## 2. Results

### 2.1. TP53 Variants in EAC Cell Lines

Three EAC cell models, OE33, OE19, and FLO1, representing distinct stages of tumor progression, were included in the study. OE33 and FLO1 cells harbor *TP53* missense mutations, classified as damaging and associated with dominant-negative and loss-of-function (DNE-LOF) effects according to the IARC *TP53* database, as detailed in Table 1 and Table S1. OE19 cells carry a *TP53* frameshift variant that introduces a premature stop codon at position 310 in the protein, likely resulting in a loss-of-function protein due to the complete loss of the tetramerization and activation domain.

**Figure 1:**
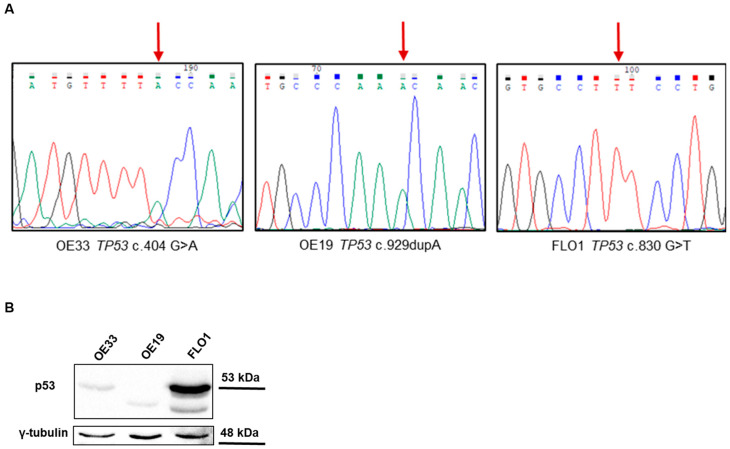
(**A**) *TP53* mutations in OE33, OE19, and FLO1 cells. The variant c.404G>A is present in OE33 cells (arrow); the variant c.929dupA is present in OE19 cells (arrow); and the variant c.830G>T is present in FLO1 cells (arrow). (**B**) Representative images of a Western blot showing p53 expression in total cell lysates derived from the three EAC cell lines (γ-Tubulin was used as endogenous control). Cropped images are shown.

The presence of *TP53* genetic alterations in our cell models was validated through Sanger sequencing, showing that all cells presented the corresponding variants as completely mutated (Figure 1A). The expression level of p53 in the different cell lines was analyzed via Western blot. Consistent with the genetic data, OE19 cells did not express the full-size protein, which was observed in the other two cell lines that carried a missense change in p53 (Figure 1B). OE33 cells exhibited lower levels of p53 protein compared to FLO1 cells, and in FLO1 cells, multiple isoforms of p53 were observed.

### 2.2. Metabolic Adaptations of EAC Cell Lines to Different Growth Environments

**Figure 2:**
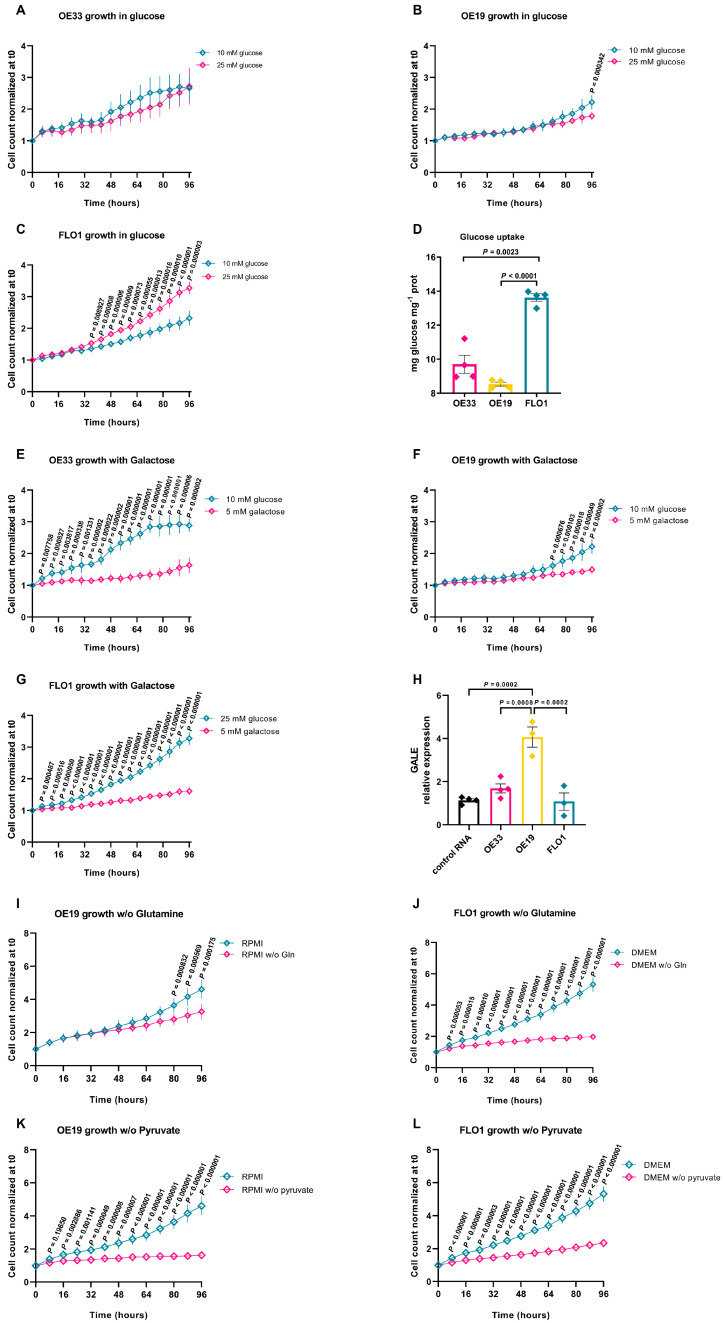
(**A**–**C**) Cell growth analysis assessed via the IncuCyte Live-Cell Assay. (**A**) OE33, (**B**) OE19, and (**C**) FLO1 cells were imaged and analyzed every 8 h over a time range of 96 h, grown in media with low glucose (10 mM, light blue) and high glucose (25 mM, magenta). Data are reported as the mean ± standard error of the mean (SEM) (at least *n* ≥ 6; multiple *t*-test, one per row, the *p*-values for each time point are reported in the graphs); (**D**) glucose uptake in OE33, OE19, and FLO1 cells. The glucose content in the growth medium was enzymatically determined based on glucose oxidase activity, and the cellular glucose uptake was calculated by subtracting the glucose measured in the tested medium from the total glucose measured in the fresh medium. The data were normalized based on cellular protein content (Brown–Forsythe and Welch ANOVA tests, OE33 vs. FLO1 *p* = 0.0023, OE19 vs. FLO1 *p* < 0.0001). Data are reported as the mean ± SEM (*n* = 4 independent experiments); (**E**–**G**) cell growth in media containing standard glucose concentrations (light blue) or 5 mM galactose (magenta). Growth of (**E**) OE33, (**F**) OE19, and (**G**) FLO1 cells was monitored every 8 h over a 96-h period using the IncuCyte system. Data are presented as the mean ± SEM (*n* ≥ 6; multiple *t*-test one per row, the *p*-values are shown in the graphs); (**H**) RT-qPCR analysis showing the relative expression of *GALE* in OE33, OE19, and FLO1 cells. Data were normalized to a commercial pooled RNA control derived from normal esophageal tissue of five independent donors. Expression values are shown relative to the internal control gene *ACTB*. Experiments were repeated at least three times (one-way ANOVA, control vs. OE19 *p* = 0.0002, OE33 vs. OE19 *p* = 0.0008, FLO1 vs. OE19 *p* = 0.0002); (**I**,**J**) effect of glutamine deprivation on the growth of (**I**) OE19 and (**J**) FLO1 cells. Cells were cultured under standard conditions (light blue) or in glutamine-free medium (magenta) for 96 h. Images were acquired every 8 h using the IncuCyte system. Data are presented as the mean ± SEM (*n* ≥ 6); statistical significance was assessed using multiple *t*-tests, one per row, *p*-values for each time point are indicated in the graphs; (**K**,**L**) impact of pyruvate deprivation on the growth of (**K**) OE19 and (**L**) FLO1 cells. Cells were cultured in either standard medium (light blue) or pyruvate-free medium (magenta) for 96 h. Images were captured every 8 h using the IncuCyte system. Data are presented as the mean ± SEM (*n* ≥ 6, multiple *t*-test, one per row, corresponding *p*-values are shown in the graphs).

To understand whether metabolic alterations influence key cellular processes such as proliferation and energy production in EAC, we first analyzed the metabolic dependence on glycolysis of the three EAC cell lines. Cells were grown for 96 h in the presence of different glucose concentrations (10 mM vs. 25 mM), and cell proliferation was monitored using the IncuCyte Live-Cell Analysis system. OE33 cells exhibited similar growth in low-glucose media (Figure 2A). At low-glucose levels, OE19 cells demonstrated a robust proliferative capacity. Even after 96 h, when glucose availability might have been expected to limit proliferation, these cells maintained an exponential growth pattern (Figure 2B). This suggests a lower reliance on glycolysis for their energy metabolism. Conversely, FLO1 cells were dependent on glucose availability, displaying significantly slower growth in media with reduced glucose concentrations vs. high-glucose medium (Figure 2C).

To assess the energetic metabolism, we used an indirect assay based on the enzyme glucose oxidase (GOX), for the estimation of glucose consumption over time. The oxidation of β-d-glucose by GOX leads to oxygen consumption and H_2_O_2_ production, thereby enabling indirect quantification of glucose utilization. Compared to OE19 and OE33 cells, FLO1 cells exhibited markedly higher glucose uptake, confirming their strong dependence on glycolysis to sustain growth and survival (Figure 2D).

To further characterize the metabolic dependency of EAC cell lines on glycolysis, we cultured cells for 96 h in a galactose-containing medium (5 mM) in the absence of glucose. Cell growth analysis in galactose revealed significant differences among the three EAC cell lines (Figure 2E–G). We observed a marked proliferation decrease in OE33 and FLO1 cells, suggesting a strong dependency on glycolysis (Figure 2E,G). In contrast, OE19 cells maintained a growth rate comparable to that observed in glucose-containing conditions, indicating a greater capacity to shift to oxidative metabolism (Figure 2F).

These findings are consistent with qRT-PCR results for *GALE* (UDP-galactose 4-epimerase) expression, a key component of the Leloir pathway, since it was significantly upregulated in OE19 cells compared to the other EAC lines and a control tissue derived from a pool of normal esophagus, suggesting that OE19 may possess a more efficient mechanism for metabolizing galactose (OE33 vs. OE19 *p* = 0.0008, OE19 vs. FLO1 *p* = 0.0002, Figure 2H).

We next evaluated whether glutamine may serve as a compensatory mechanism to sustain proliferation in the glycolysis-dependent FLO1 cells vs. OE19 cells. In glutamine-free medium, OE19 cells exhibited only a partial slowdown in growth, whereas FLO1 cells underwent a complete growth arrest as early as 24 h, indicating a pronounced sensitivity to the absence of this amino acid (Figure 2I,J). We then evaluated the impact of pyruvate deprivation on cell growth. As shown in Figure 2K,L, pyruvate removal from the culture medium markedly impaired the proliferation rate of OE19 cells, indicating a strong dependency on oxidative metabolism, whereas FLO1 cells exhibited a milder response, suggesting that they are less dependent on pyruvate oxidation.

### 2.3. EAC Cell Lines Exhibit Different Levels of Lactate Production and Oxygen Consumption

**Figure 3:**
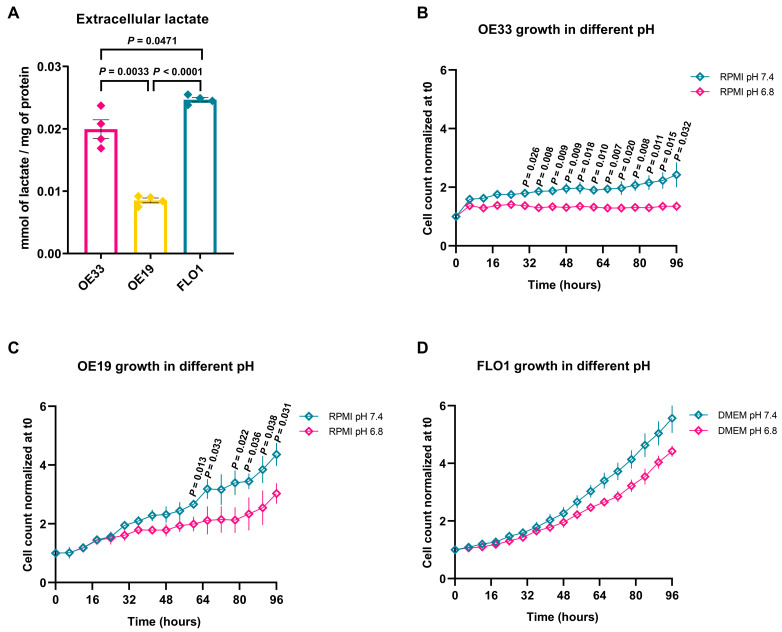
(**A**) Extracellular lactate content determination using HPLC. The extracellular lactate content in culture cell medium was quantified after 48 h of cell growth. Data are reported as the mean ± SEM (*n* = 4 independent experiments; Brown–Forsythe and Welch ANOVA tests, OE33 vs. FLO1 *p* = 0.0471, OE33 vs. OE19 *p* = 0.0033, OE19 vs. FLO1 *p* < 0.0001); (**B**–**D**) effect of acidic pH on cell growth. Growth of (**B**) OE33, (**C**) OE19, and (**D**) FLO1 cells in standard medium (pH = 7.4, light blue) and acidic medium (pH = 6.8, magenta) was monitored over 96 h using the IncuCyte system, with images taken every 8 h. Data are presented as the mean ± SEM (*n* ≥ 6). Statistical significance was assessed using multiple *t*-tests (one per row); *p*-values are indicated in the graphs.

To further investigate the metabolic profile of the three EAC cell lines, we measured extracellular lactate levels via High-Performance Liquid Chromatography (HPLC). Lactate quantification is used as a marker of the predominant metabolic pathway, as high extracellular concentrations of this metabolite indicate a preference for aerobic glycolysis [24]. FLO1 cells produced the highest levels of extracellular lactate, indicating a strong dependence on aerobic glycolysis. OE33 cells also exhibited elevated lactate levels. In contrast, OE19 cells generated significantly less lactate, in accordance with a reduced dependence on aerobic glycolysis and a preference for oxidative metabolism (Figure 3A).

In solid tumors, extracellular pH (pHe) tends to become more acidic due to the intense glycolytic activity of cancer cells, which leads to lactate accumulation [25]. To assess the impact of an acidic microenvironment on cell proliferation, the cell lines were cultured for 96 h at pH 6.8 vs. pH 7.4 (physiological conditions). OE33 cells were more sensitive to the acidic environment and showed almost complete growth arrest over time (Figure 3B). OE19 cells exhibited a significant decrease in growth, but proliferation still persisted (Figure 3C). Notably, FLO1 cells were less sensitive to acidity, maintaining a proliferation rate similar to that observed at physiological pH (Figure 3D).

### 2.4. OE19 Cells Reveal an Energy Imbalance with Increased Reactive Oxygen Species Production

**Figure 4:**
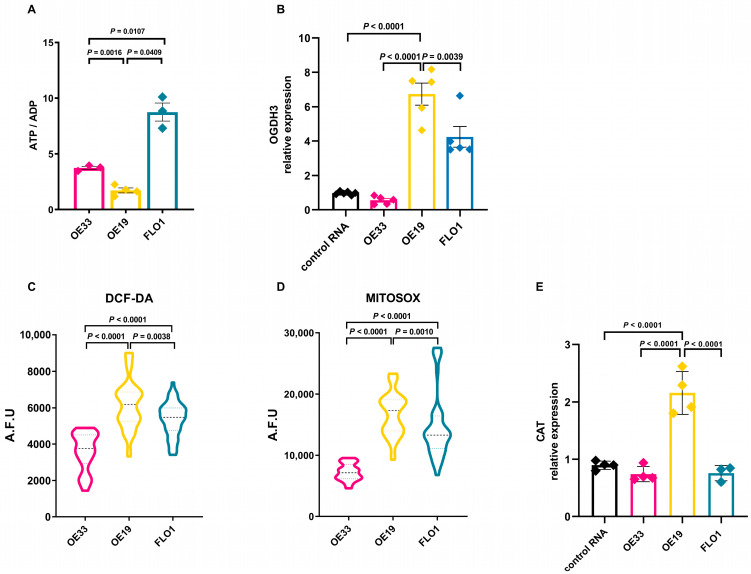
(**A**) ATP/ADP ratio in cellular extracts (*n* ≥ 3; Brown–Forsythe and Welch ANOVA tests, OE33 vs. FLO1 *p* = 0.0107, OE33 vs. OE19 *p* = 0.0016, OE19 vs. FLO1 *p* = 0.0409). (**B**) RT-qPCR analysis demonstrating the relative expression of *OGDH3* in OE33, OE19, and FLO1 cells. Data were normalized using a commercially available RNA control. *ACTB* was used as the endogenous reference gene. The experiments were conducted at least in triplicate (One-way ANOVA, control vs. OE19 *p* < 0.0001, OE33 vs. OE19 *p* < 0.0001, and FLO1 vs. OE19 *p* = 0.0222). (**C**) Violin plot showing intracellular ROS levels measured based on DCF-DA fluorescence (arbitrary fluorescence units, A.F.U.) in OE33, OE19, and FLO1 cells. At least 40 individual measurements were performed per cell line (Mann-Whitney test, OE33 vs. FLO1 *p* < 0.0001, OE33 vs. OE19 *p* < 0.0001, and OE19 vs. FLO1 *p* = 0.0038); (**D**) mitochondrial oxidative stress determination in OE33, OE19, and FLO1 cells using MitoSOX. Each cell line was analyzed with no fewer than 40 replicates (*n* = 6, Mann–Whitney test, OE33 vs. FLO1 *p* < 0.0001, OE33 vs. OE19 *p* < 0.0001, and OE19 vs. FLO1 *p* = 0.0010). (**E**) RT-qPCR analysis showing the relative expression of *CAT* in OE33, OE19, FLO1, and a control RNA (commercially available). Expression levels were normalized to the internal reference gene *ACTB* (*n* ≥ 3, one-way ANOVA, control vs. OE19 *p* < 0.0001, OE33 vs. OE19 *p* < 0.0001, OE19 vs. FLO1 *p* < 0.0001).

The ATP/ADP ratio is a key parameter of cellular energy metabolism that determines the free-energy change for ATP hydrolysis and is also a sensitive indicator of energy consumption and changes in cellular energy status. Therefore, we measured the ATP/ADP ratio in our cell models, observing that OE19 cells exhibited the lowest ATP/ADP ratio, whereas FLO1 cells showed the highest ratio, suggesting markedly elevated energy production (Figure 4A).

We next analyzed the expression levels of oxoglutarate dehydrogenase (*OGDH*), which catalyzes the oxidation of 2-oxoglutarate (alpha-ketoglutarate) to succinyl-CoA and CO_2_ in the tricarboxylic acid (TCA) cycle. OE19 cells exhibited the highest *OGDH* expression compared to the other cell lines, supporting oxidative metabolism (OE19 vs. OE33 *p* = 0.0016, OE19 vs. FLO1 *p* = 0.0409; Figure 4B).

To investigate the redox status of these cell lines, we assessed the production of reactive oxygen species (ROS), using MitoSOX, a specific dye for mitochondrial superoxide, and DCF-DA (2′,7′-dichlorofluorescin diacetate), a general indicator of total intracellular ROS production. Fluorescence levels of both probes were significantly higher in OE19 cells (FLO1 vs. OE19, *p* = 0.0010; OE33 vs. OE19, *p* < 0.0001) (Figure 4C,D), indicating increased oxidative stress in these cells. The increase in ROS production was accompanied by significantly higher expression of the ROS-detoxifying enzyme *CAT* in OE19 cells compared to normal esophageal tissue and the other cell lines (OE19 vs. OE33, *p* < 0.0001, OE19 vs. FLO1 *p* < 0.0001) (Figure 4E). *CAT* encodes catalase, a crucial antioxidant enzyme that mitigates oxidative stress by decomposing cellular hydrogen peroxide into water and oxygen, thereby preventing its cytotoxic accumulation [26]. The expression levels of *SOD1* and *SOD2*, encoding for different superoxide dismutases, were also evaluated, showing that *SOD1* expression was highest in FLO1 cells, whereas *SOD2* expression was more pronounced in OE33 cells (Figure S2).

### 2.5. TP53 Restoration Reduces Extracellular Lactate Levels

**Figure 5:**
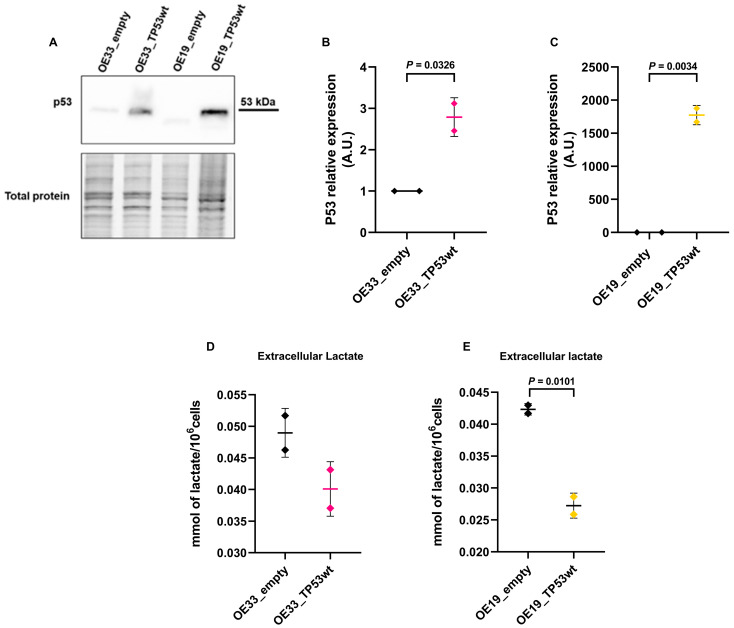
(**A**) Western blot showing p53 protein levels in OE33 and OE19 cells 48 h after transfection with the empty vector (pcDNA3.1) or wild-type *TP53*-expressing plasmid (pcDNA3.1_TP53wt). Total protein staining was used as a loading control. Cropped images are shown. (**B**,**C**) Densitometric quantification shows increased p53 expression in both OE33 and OE19 cells after TP53wt transfection. Data represent the mean ± SD (*n* = 2, unpaired *t* test, OE33_empty vs. OE33_TP53wt *p* = 0.0326, OE19empty vs. OE19_ TP53wt *p* = 0.0034). (**D**,**E**) Extracellular lactate levels measured via HPLC in culture media of (**D**) OE33 and (**E**) OE19 cells 48 h post-transfection. Lactate concentrations were normalized to cell number. A significant reduction in lactate was is observed in OE19 cells transfected with wild-type *TP53* compared to the empty vector controls. Data represent the mean ± SD (*n* = 2, Unpaired *t* test, OE19empty vs. OE19_ TP53wt *p* = 0.0101).

To explore how *TP53* restoration might affect the cells’ metabolism in intermediate tumor stages, i.e., in OE33 and OE19 cells, we transiently re-expressed *TP53* with a plasmid encoding wild-type *TP53* cDNA and compared them with the corresponding cells transfected with the empty plasmid. Western blot analysis confirmed the overexpression of p53 protein in both cells after transfection with the *TP53* construct compared to their respective controls (Figure 5A). Re-expression of *TP53* resulted in a statistically significant increase in p53 in OE33 (OE33_empty vs. OE33_TP53wt *p* = 0.0326) (Figure 5B) and OE19 cells (OE19_empty vs. OE19_TP53wt *p* = 0.0034) (Figure 5C). Extracellular lactate levels were measured in the culture medium of OE33 and OE19 cells 48 h post-transfection. In both cell lines, re-expression of wild-type *TP53* led to a reduction in extracellular lactate secretion compared to empty vector controls (Figure 5D), which reached statistical significance in OE19 cells (OE19_empty vs. OE19_TP53wt *p* = 0.0101) (Figure 5E).

### 2.6. Transcriptomic Analysis Reveals TP53 Mutation-Dependent Metabolic Reprogramming in EAC

**Figure 6:**
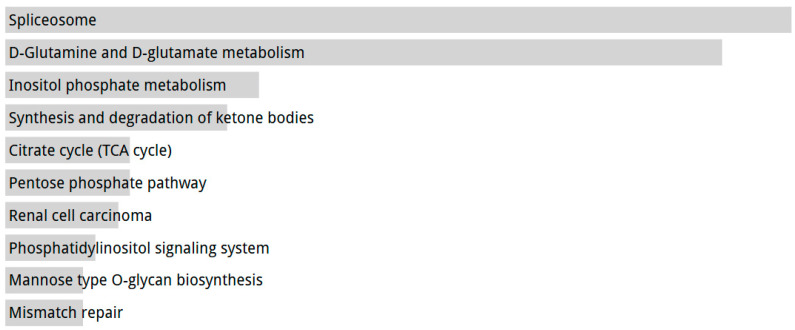
KEGG pathway enrichment of differentially expressed genes between TP53-mutated and wild-type EAC samples from TCGA. Bar plot representing the top significantly enriched KEGG pathways identified in *TP53*-mutated vs. *TP53*-wild-type EAC tumors, based on RNA-seq data from TCGA. Pathways are ranked by −log10 (adjusted *p*-value). Full gene lists are provided in Table S2.

To determine whether the metabolic differences observed in our cell models are also present in EAC patients with *TP53* mutations, we analyzed publicly available transcriptomic data from The Cancer Genome Atlas (TCGA) EAC cohort. Notably, *TP53* is the gene most frequently mutated in this cohort of patients with EAC (Figure S1). Gene expression profiles of *TP53*-mutated and *TP53*-wild-type tumors were compared, and differentially expressed genes were subjected to KEGG pathway enrichment analysis. This analysis revealed significant overrepresentation of several metabolic pathways in *TP53*-mutated tumors, including the citrate cycle (TCA cycle), glutamine and glutamate metabolism, inositol phosphate metabolism, and the pentose phosphate pathway (Figure 6, Table S2). These findings indicate that *TP53*-mutated EACs exhibited a distinct metabolic signature, consistent with the functional reprogramming observed in vitro.

## 3. Discussion

EAC represents a growing public health problem in Western countries, due to its increasing incidence and poor prognosis [1]. Five-year survival rates remain low, highlighting the need for a better understanding of the molecular mechanisms underlying this malignancy [27]. Despite advances in omics technologies that have improved our understanding of the genetic and epigenetic landscape in EAC, translating these findings into clinical practice remains a challenge. There is growing interest in identifying molecular and metabolic biomarkers, exploring innovative diagnostic approaches, and developing personalized therapeutic targets to improve treatment efficacy.

Therefore, we undertook a metabolic profiling of EAC cellular models harboring different *TP53* mutations and corresponding to distinct stages of tumor progression.

The OE33 cell line, representative of an early stage of tumor progression, harbors a missense mutation within the DNA-binding domain of *TP53*, previously classified as potentially pathogenic [28]. This alteration affects a cysteine residue that, together with three other cysteines, coordinates the formation of a pocket capable of binding arsenic trioxide (ATO) [29]. Notably, ATO has been shown to trigger apoptosis in cancer cells carrying *TP53* mutations [30,31]. OE33 cells showed a glycolytic phenotype, also observed in FLO1 cells.

In FLO1 cells, the missense *TP53* mutation affects the amino acid Cys277, which, along with Lys120 and Arg280, is essential for DNA interaction through the formation of hydrogen bonds with the minor groove. The substitution of Cys277 with Phenylalanine, an aromatic amino acid, impairs this interaction and hinders the tumor-suppressive function of p53 [29]. FLO1 cells exhibited a decreased proliferation rate when cultured in low glucose conditions, which was more evident when galactose was used as the carbon source instead of glucose. Unlike glucose, galactose is metabolized more slowly through the Leloir pathway, which converts it into glucose-6-phosphate before entering glycolysis [32]. Due to the reduced efficiency of this pathway, cells cultured in galactose must rely more heavily on mitochondrial oxidative phosphorylation (OXPHOS) to meet their energy demands [33,34].

Indeed, the analysis of the bioenergetic status revealed that FLO1 cells were highly dependent on glycolysis to support energy production. The significantly higher ATP/ADP ratio observed in FLO1 cells compared to OE19 suggests more sustained and rapid ATP turnover, which is consistent with a glycolytic phenotype. Although glycolysis is less efficient in terms of ATP yield per glucose molecule, its markedly accelerated rate can lead to a favorable ATP/ADP ratio, ensuring adequate energy availability even in the absence of robust oxidative phosphorylation [35]. FLO1 cells showed pronounced growth arrest in glutamine deprivation. Glutamine, beyond its central role in various metabolic processes, is critical for replenishing intermediates of the Krebs cycle. Additionally, glutamine provides nitrogen essential for synthesizing alanine, aspartate, and serine—amino acids crucial for cancer cell proliferation [36]. Our findings support the concept that in highly proliferating FLO1 cells, glutamine is necessary to sustain metabolism when pyruvate derived from glucose is converted into lactate and secreted [37].

OE19 cells carry a frameshift mutation in *TP53* and *ERBB2* amplification. Interestingly, *TP53* mutations are associated with increased mortality in patients with HER2-overexpression [38], a receptor kinase encoded by the *ERBB2* gene. The *ERBB2* amplification reported in the OE19 cell line [18] might contribute to the observed metabolic features in these cells, since HER2 expression has been associated with enhanced oxidative phosphorylation activity and elevated mitochondrial ROS production [39]. Notably, mitochondrial respiratory metabolism seems to be the preferred metabolic pathway in these cells. Indeed, the absence of pyruvate in the culture medium also induced a drop in the growth of OE19 cells. Pyruvate plays a key role in supporting the proliferation of different types of cancer. Upon conversion to acetyl-CoA, it fuels the TCA cycle and supports mitochondrial respiratory metabolism [40]. The strong reliance of OE19 cells on pyruvate oxidation was further confirmed by *OGDH* overexpression, which encodes for one subunit of the 2-oxoglutarate dehydrogenase complex enzyme involved in the oxidative decarboxylation of alpha-ketoglutarate to succinyl-CoA in the TCA cycle.

The recent literature [41,42,43,44] supports the notion that mitochondrial insufficiency in tumors such as colorectal, lung, and breast cancers is closely linked with dysregulation of deuterium content in metabolic water. In this scenario, the deutenome profiling [45] may serve as a diagnostic strategy, offering a novel tool through which to interpret metabolic changes in epithelial malignancies.

The different metabolic requirements of the EAC cell lines used in this study might pave the way to identify specific metabolic vulnerabilities that could be translated into therapeutic strategies for EAC treatment. Tumor cells with a strong dependence on glycolysis, such as OE33 and FLO1, may be particularly sensitive to therapies targeting this metabolic pathway, such as the use of 2-deoxyglucose (2-DG) or the inhibition of lactate dehydrogenase [46]. Furthermore, the marked glutamine dependency of FLO1 cells suggests that the deprivation of this amino acid, either through the use of structural analogs (e.g., DON-6-diazo-5-oxo-L-norleucine) [47] or by inhibiting glutaminase (GLS) [48], might represent another effective therapeutic approach. Although glutamine is essential for organisms and there is evidence that supports the beneficial role of glutamine in cancer state and cardiometabolic disorders [49], dysregulation of glutaminase and glutamine synthetase promotes the anabolic adaptation of tumors. Specific drugs targeting key enzymes of glutamine metabolism have been able to eliminate some neoplasms [36,50]. However, induced adaptive metabolic resistance must be considered in designing synergistic combination therapies as anticancer tools to overcome both cancer growth and resistance mechanisms [51].

Finally, OE19 cells are characterized by a greater reliance on mitochondrial oxidative metabolism and a state of elevated oxidative stress. Targeting OGDH, whose expression is upregulated in different types of cancer to enhance mitochondrial metabolism, might represent a specific approach to fight the progression of EAC cases showing a similar mutation pattern [52]. Moreover, the combined use of these molecules with current drugs and therapeutic strategies could prove particularly effective, especially for more aggressive EAC cases.

Our in vitro findings were corroborated by the analysis of TCGA data, which revealed that *TP53*-mutated EAC tumors are enriched in metabolic pathways such as the TCA cycle, pentose phosphate pathway, and glutamate metabolism. These results suggest that *TP53* mutations are also associated with broader metabolic reprogramming in patient-derived tumors, highlighting the clinical relevance of targeting specific bioenergetic vulnerabilities in EAC, including the assessment of tumor microenvironment and extracellular metabolites.

The metabolic characterization in this study was conducted using a limited number of established EAC cell lines, which may not fully reflect the genetic and metabolic heterogeneity of patient-derived tumors. Moreover, normal control cells for esophageal adenocarcinoma are scarcely available, since the origin of this neoplasm is still debated, in contrast with esophageal squamous cancer, derived from the squamous epithelia lining the esophagus. These in vitro findings should be corroborated with additional ex vivo models [53]. Nonetheless, the observed metabolic alterations underscore the importance of considering tumor-specific metabolic features when selecting therapeutic strategies, with the aim of maximizing treatment efficacy while minimizing systemic toxicity.

## 4. Materials and Methods

### 4.1. PCR and Sanger Sequencing

PCR amplification was performed on genomic DNA extracted from cell lines with the DNeasy Blood & Tissue Kit according to the manufacturer’s instructions (Qiagen, Hilden, Germany). Primer pairs were designed with Primer3 software (v4.1.0) and include the following: *TP53* exon 4 Fw-GATGCTGTCCGCGGACGATAT; *TP53* exon 4 Rv-CGTGCAAGTCACAGACTTGGC; *TP53* exon 7 Fw:-GGCTCTGACTGTACCACCAT; *TP53* exon 7 Rv-TGATGATGGTGAGGATGGGC; *TP53* exon 8 Fw-GGGACAGGTAGGACCTGATTT; *TP53* exon 9 Rv-TCAGGCAAAGTCATAGAACCA.

The following PCR conditions were used: KAPA HiFi HotStart (Hoffmann-La Roche, Basel, Switzerland), MgCl_2_, 20 ng gDNA, dNTPs. The PCR cycles are as follows: activation at 95 °C 3′ and 40 cycles of 95 °C for 15″, 60 °C for 15″, 72 °C for 30″, and 72 °C for 1′. Following purification on the 96-well multiscreen PCR system (Millipore, Thermo Fisher Scientific, Waltham, MA, USA), PCR products were sequenced with the BigDye v1.1 kit and subsequently analyzed on a 3730 DNA Analyzer (Thermo Fisher Scientific). Electropherograms were visualized using Chromas 2.0 (Chromas, Technelysium, South Brisbane, Australia).

### 4.2. Western Blot Analysis

Cell lysis and Western blot analysis were performed according to Diquigiovanni et al. [54]. The primary anti-p53 antibody (sc-126, Santa Cruz Biotechnology, Dallas, TX, USA) was used at a dilution of 1:500 in a 1% Casein-TBS (Bio-Rad, Hercules, CA, USA) and incubated at 4 °C for 16 h. The secondary anti-mouse antibody (Sigma-Aldrich, St. Louis, MO, USA) was applied at a dilution of 1:5000 in 1% Casein-TBS (Bio-Rad) with 1 h incubation at room temperature. Protein bands were visualized using the Clarity ECL system (Bio-Rad) and detected with the ChemiDoc™ XRS+ system (Bio-Rad). Images were acquired and analyzed using Image Lab software v5.1 (Bio-Rad).

### 4.3. Cell Cultures and Experimental Conditions

OE19 (ECACC: 96071721) and OE33 (ECACC: 96070808) were cultured in Roswell Park Memorial Institute (RPMI)-1640 medium (EuroClone, Pero, Italy) supplemented with 10% fetal bovine serum, 100 U/mL penicillin, and 100 μg/mL streptomycin. FLO1 cells (ECACC: 11012001) were cultured in Dulbecco’s Modified Eagle Medium (DMEM) (EuroClone) supplemented with 10% (*v*/*v*) FBS, 100 U/mL penicillin, 100 μg/mL streptomycin, and 2 mM L-glutamine (Sigma-Aldrich). All cells were cultured at 37 °C in a 5% CO_2_ incubator. All cells tested negative for mycoplasma, as previously reported [54]. For experimental procedures, cell lines were cultured in their respective growth media under various experimental conditions for a total duration of 96 h. Cells were seeded in 96-well plates at a density of 1000 cells per well 24 h prior to treatment. The growth media were modified as follows: (i) 10 mM glucose, (ii) 25 mM glucose, (iii) DMEM or RPMI containing 5 mM galactose, (iv) glutamine-free medium, and (v) pyruvate-free medium. To obtain media at a specific pH level (pH 6.8), drops of 0.1 M HCl (Sigma-Aldrich) were added gradually to the media. pH was monitored using a pH meter (ORION 420A, Thermo Scientific).

Cell proliferation across the different treatment conditions was monitored in real time for 96 h using the IncuCyte ^®^ Live-Cell Analysis System (Sartorius, Göttingen, Germany) to continuously monitor and quantify cell proliferation, capturing images and calculating cell confluence at regular intervals (6/8 h). Data were collected using IncuCyte v.2022B Rev2 GUI software, and post-acquisition analysis of proliferation data was conducted using GraphPad Prism v8.0 (GraphPad Software Inc., La Jolla, CA, USA).

### 4.4. Glucose Oxidase Assay

Cells were seeded at a concentration of 1.5 × 10^5^ and 0.9 × 10^5^ in a 6-well plate. After 48 h, 50 µL of medium was collected and the glucose level was assessed using an enzymatic assay based on β-d-glucose:oxygen 1-oxidoreductase (GOX) activity, which catalyzes the oxidation of β-d-glucose to d-gluconolactone, converting molecular oxygen to hydrogen peroxide [55]. The reaction was carried out in 1.6 mL of 50 mM sodium acetate buffer in the presence of 57 µg/mL GOX, pH 5.1, 30 °C. The reaction was initiated by the addition of 50 µL of cell culture medium. The oxygen consumption rate was monitored for 30 s in a thermostatically controlled oxygraphic chamber (Yellow Springs Instrument YSI 53, Dayton, OH, USA). A titration curve with glucose standards was used for calibration. Glucose uptake was calculated as the difference between glucose in the medium and fresh medium, normalized to protein content.

### 4.5. Quantitative Reverse Transcriptase Real-Time PCR (qRT-PCR)

Total RNA was extracted from 1.5 × 10^5^ OE19, OE33, and FLO1 cells using the RNeasy Plus Mini Kit (Invitrogen, Thermo Fisher Scientific, Waltham, MA, USA), and RNA concentration and quality were verified with a NanoDrop 2000 spectrophotometer (Thermo Fisher Scientific). Approximately 500 ng of RNA was used for cDNA preparation using the SuperScript ™ VILO ™ Master Mix synthesis Kit for RT-qPCR with *DNase I* (Thermo Fisher Scientific) in a final volume of 20 μL. qRT-PCR was performed in triplicate for each point using the SYBR Green kit (Qiagen) in a StepOne Plus Real Time PCR system (Thermo Fisher Scientific). *ACTB* (human actin-beta gene) was used as an endogenous gene normalizer. Expression in the cell lines was compared to a commercial pool of normal esophageal RNAs from 5 different donors (BioChain, Newark, CA, USA), expressed as a fold change using the 2^−ΔΔCt^ method [56]. Human-specific primers for the genes of interest include the following: *hACT* Fw-CCTGGCACCCAGCACAAT; *hACT* Rv-GGGCCGGACTCGTCATACT; *GALE* Fw-AAATGATCCGGGACCTGTGC; *GALE* Rv-ATCGCCACCTGGGAGACATA; *OGDH3* Fw-GCCCAACGTGGACAAGCTGGT; *OGDH3* Rv-GGGCATAGAACCCCACGTTTGAAGA; *CAT* Fw-GTGCGGAGATTCAACACTGCCA; *CAT* Rv-CGGCAATGTTCTCACACAGACG.

### 4.6. Transient Transfection of Wild-Type TP53

Of note, 3 × 10^5^ cells of each EAC cell line were seeded 24 h prior to transfection with either a plasmid encoding wild-type *TP53* cDNA (pcDNA3.1_TP53wt) or an empty vector (pcDNA3.1), using Lipofectamine 3000 (Thermo Fisher Scientific), according to the manufacturer’s instructions. Cells were harvested and washed twice with PBS 48 h after transfection. Western blot and lactate analyses were performed as described above.

### 4.7. Lactate Quantification

Extracellular lactate was measured as described [57]. Briefly, 1.0 × 10^5^ cells were seeded in 6-well plates and cultured for 48 h in complete medium. Then, the culture medium was collected and diluted (1:3) in the mobile phase (50 mM KH_2_PO_4_, pH 2.9) and centrifuged at 14,000× *g* for 5 min at 4 °C. Samples were analyzed with an HPLC system using a CI8 column (Agilent ZORBAX SB-Phenyl, 5 µm, 250 × 4.6 mm). Lactate was detected at 210 nm using a UV detector and quantified with ChemStation software (v.LTS 01.11), based on a calibration curve. Peak areas were normalized based on protein content, determined using the DC Protein Assay (Bio-Rad).

### 4.8. ATP and ADP Determination

Nucleotides were extracted and detected following the method of Jones [58], using a Kinetex C18 column (250 × 4.6 mm, 100 Å, 5 µm; Phenomenex, Torrance, CA, USA). Absorbance (260 nm) was monitored with a photodiode array detector (Agilent 1100 series system; Agilent Technologies, Santa Clara, CA, USA). Nucleotide peaks were identified through comparison and coelution with standards and quantification by peak area measurement compared with standard curves.

### 4.9. Radical Oxygen Species and Mitochondrial Anion Superoxide Measurement

ROS production was evaluated using fluorescent probes H_2_DCFDA (2′,7′-dichlorodihydrofluorescein diacetate; Thermo Fisher Scientific) and MitoSOX ™ Red (Molecular Probes, Invitrogen, Eugene, OR, USA). Cells (0.2 × 10^5^/well) were seeded in 96-well black plates (OptiPlate Black, PerkinElmer, Waltham, MA, USA) in complete medium. After 24 h, general ROS levels were assessed by incubating cells with 10 µM H_2_DCFDA in complete medium for 30 min. Following two washes with HBSS, fluorescence was measured at λ_ex_  =  485 nm and λ_em_  =  535 nm using a multiplate reader (EnSpire, PerkinElmer). Mitochondrial anion superoxide production was determined by incubating the cells with 5 µM MitoSOX ™ Red in complete medium for 30 min. After washing with HBSS, fluorescence was measured at λ_ex_  =  510 nm and λ_em_  =  580 nm. For both experiments, fluorescence emission was normalized based on protein content determined using the DC protein assay (Bio-Rad).

### 4.10. In Silico Analysis of TCGA Transcriptomic Data

Transcriptomic data (RNA-seq) from a cohort of 186 EAC samples were obtained from the TCGA using the cBioPortal platform (https://www.cbioportal.org, accessed on 25 June 2025). Samples were stratified based on *TP53* mutation status: 156 tumors carried *TP53* mutations, while 30 were *TP53* wild-type. Differentially expressed genes between the two groups were identified using the integrated comparison tools provided by cBioPortal. A total of 5717 genes were identified as significantly differentially expressed (adjusted *p*-value < 0.05). The resulting gene list was submitted to KEGG pathway enrichment analysis using the Enrichr tool (https://maayanlab.cloud/Enrichr/, accessed on 25 June 2025) and the KEGG 2021 Human database.

### 4.11. Statistical Analysis

The Mann–Whitney test, Brown–Forsythe and Welch tests, Multiple *t*-test, or one-way analysis of variance with multiple comparisons test were performed as reported in the Results sections and figure legends, using GraphPad Prism v8.0 software (GraphPad Software Inc.). *p*-values < 0.05 were considered statistically significant. The in vitro data are representative of at least 3 biologically independent experiments. All data collected are reported as the mean ± standard error of the mean.

## 5. Conclusions

Our study reveals distinct metabolic dependencies across EAC cell models harboring different *TP53* mutations, reflecting the molecular and phenotypic heterogeneity observed in patients. OE19 cells rely on mitochondrial oxidative phosphorylation, potentially influenced by *ERBB2* amplification. OE33 cells display an intermediate metabolic profile, linking early-stage tumorigenesis with glycolytic reprogramming. FLO1 cells, representative of advanced-stage EAC, exhibit a pronounced glycolytic dependence. These findings suggest that *TP53* mutation type might shape the metabolic landscape of EAC tumor progression. By uncovering specific metabolic dependencies, we propose a rationale for tailoring metabolic interventions to individual tumor profiles. Ultimately, integrating metabolic phenotyping into molecular stratification could guide the development of more effective, personalized therapeutic strategies for EAC.

## Figures and Tables

**Table 1:** Type of change in the coding region of TP53 (NM_000546) and associated genomic metrics in EAC cell lines.

Cell Line	Mutation	Variant ID	Position (GRCh38)	CADD	MAF	IARC Classification	Functional Impact
OE33	*TP53* c.404 G>A, p.Cys135Tyr	rs587781991	chr17:7675208	28.2	1.2 × 10^−6^	Damaging	DNE-LOF
OE19	*TP53* c.929dupA, p.Asn310Lysfs*27	n.a.	chr17:7673598	n.a.	n.a.	n.a.	n.a.
FLO1	*TP53* c.830 G>T, p.Cys277Phe	rs763098116	chr17:7673790	27.1	6.2 × 10^−7^	Damaging	DNE-LOF

MAF = Minor Allele Frequency (the reported values refer to the global allele frequency of the variant according to the gnomAD database, version v4.1.0, accessed on 29 April 2025). CADD = Combined Annotation Dependent Depletion score (obtained from gnomAD v4.1.0, accessed on 29 April 2025). Genomic positions refer to the human reference genome GRCh38 (hg38). IARC *TP53* mutation classifications were obtained from the IARC *TP53* Database accessed on 25 June 2025 (https://tp53.cancer.gov/). DNE = dominant-negative effect. LOF = loss-of-function. n.a. = not available.

## Data Availability

All data are available from the authors upon request, according to section “MDPI Research Data Policies” at https://www.mdpi.com/ethics (accessed on 14 July 2025).

## Supplementary Materials

The following supporting information can be downloaded at https://www.mdpi.com/article/10.3390/ijms26146869/s1.

## Abbreviations

The following abbreviations are used in this manuscript:

2-DG	2-deoxyglucose
ACTB	Human actin-beta
ATO	Arsenic trioxide
BFB	Breakage–fusion–bridge
CADD	Combined annotation-dependent depletion
CAT	Catalase
EAC	Esophageal Adenocarcinoma
EACSGE	Esophageal Adenocarcinoma Study Group Europe
GALE	UDP-galactose 4-epimerase
GC-MS	Gas chromatography mass spectrometry
GLS	Glutaminase
GOX	Glucose Oxidase
HPLC	High-Performance Liquid Chromatography
MAF	Minor allele frequency
NMR	Nuclear Magnetic Resonance
OGDH	Oxoglutarate dehydrogenase
OXPHOS	Oxidative phosphorylation
ROS	Reactive Oxygen Species
SOD1	Superoxide Dismutase 1
SOD2	Superoxide Dismutase 2
TCA	Tricarboxylic acid
TCGA	The Cancer Genome Atlas
