# Supplementary material for: Metabolic Profiling of Distinct TP53-Mutant Esophageal Adenocarcinoma Models Reveals Different Bioenergetic Dependencies
Source: Int J Mol Sci. 2025 Jul 17;26(14):6869. doi: 10.3390/ijms26146869 (PMC12295312; doi:10.3390/ijms26146869)

Supplementary Figure S1. Bar chart showing the most frequently mutated genes in EAC based on data from TCGA (https://www.cancer.gov/ccg/research/genome-sequencing/tcga). *TP53* is the most frequently mutated gene, found in over 80% of cases.

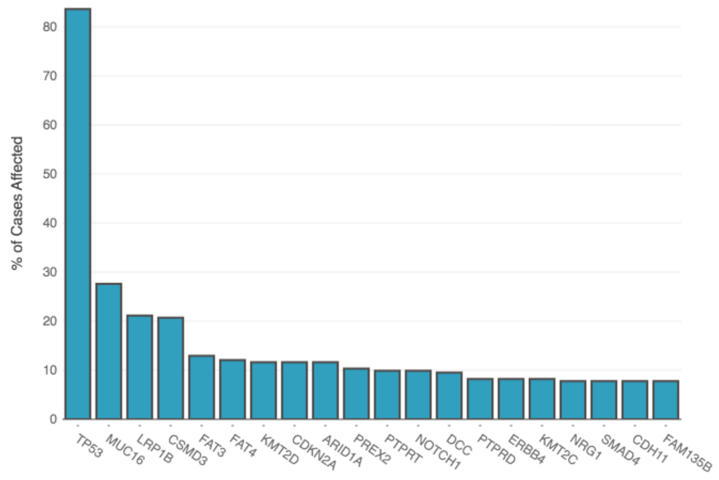

Supplementary Figure S2. RT-qPCR analysis of (**A**) *SOD1* and (**B**) *SOD2* transcript levels in OE33, OE19 and FLO1 cells. Gene expression was normalized to a commercial pooled RNA control derived from normal esophageal tissue of five independent donors. *ACTB* was used as internal control gene (*SOD1*: One-way ANOVA, control *vs.* FLO1 $P = 0.0016$, OE33 *vs.* FLO1 $P = 0.0192$, FLO1 *vs.* OE19 $P = 0.01$; *SOD2* One-way ANOVA, control *vs.* OE33 $P = 0.0005$, OE33 *vs.* OE19 $P < 0.0001$, FLO1 *vs.* OE19 $P = 0.0094$, FLO1 *vs.* OE33 $P = 0.0072$).

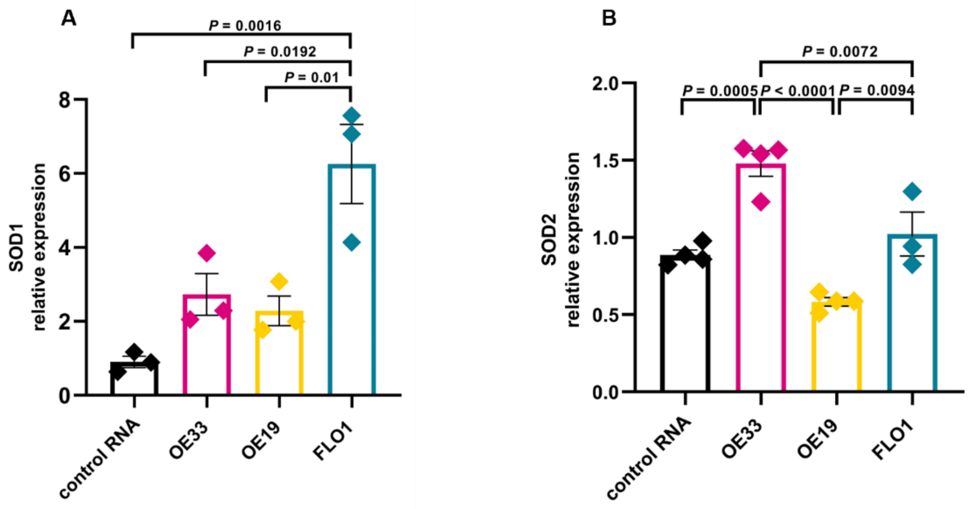

Supplement: Supplementary file 1 [file ijms-26-06869-s001.zip › Supplementary Figures.pdf]
